# Burden with No Benefit: Prior Authorization in Congenital Cardiology

**DOI:** 10.1007/s00246-023-03255-1

**Published:** 2023-09-26

**Authors:** Brian S. Marcus, Neha Bansal, Joshua Saef, Christina Fink, Angira Patel, Katherine D. Shaffer, John E. Mayer, Jonathan N. Johnson, Kenneth Shaffer, Devyani Chowdhury

**Affiliations:** 1 Pediatric Cardiology, Yale School of Medicine, 205 Church Street, New Haven, CT USA; 2 Pediatric Critical Care, The Medical College of Wisconsin, Milwaukee, United States; 3 Pediatric Cardiology, Mount Sinai Kravis Children’s Hospital, New York, NY USA; 4 Philadelphia Adult Congenital Heart Center, University of Pennsylvania, Philadelphia, PA USA; 5 Heart Institute, Joe DiMaggio Childrens Hospital, Hollywood, Florida USA; 6 Childrens Institute Department of Heart, Vascular & Thoracic, Division of Cardiology & Cardiovascular Medicine, Cleveland Clinic Children’s Hospital, Cleveland, OH USA; 7 Ann & Robert H. Lurie Childrens Hospital, Chicago, IL USA; 8 Northwestern University Feinberg School of Medicine, Chicago, IL USA; 9 Texas Tech University Health Sciences Center, Jerry H. Hodge School of Pharmacy, Abilene, TX USA; 10 Department of Cardiac Surgery, Harvard Medical School, Boston Children’s Hospital, Boston, MA USA; 11 Division of Pediatric Cardiology, Department of Pediatric and Adolescent Medicine, Mayo Clinic, Rochester, MN USA; 12 Dell Medical School, University of Texas, Austin, TX USA; 13 Pediatric and Congenital Cardiology Associates / Pediatrix Cardiology, Austin, TX USA; 14 Nemours Cardiac Center, Wilmington, DE USA; 15 Cardiology Care for Children, Lancaster, PA USA

**Keywords:** Prior authorization, Reform, Pediatric cardiology, Congenital heart disease

## Abstract

Prior authorization is a process that health insurance companies use to determine if a patient’s health insurance will cover certain medical treatments, procedures, or medications. Prior authorization requests are common in adult congenital and pediatric cardiology (ACPC) due to need for advanced diagnostics, complex procedures, disease-specific medications, and the heterogeneity of the ACPC population. Prior authorizations in ACPC are rarely denied, but nonetheless, they are often accompanied by significant administrative burden on clinical care teams and delays in patient care. Prior authorizations have been implicated in worsening care inequities. The prior authorization process is insurer specific with differences between commercial and public insurers. Prior authorization rejections were previously found to be more common for women, racial minorities, those with low education, and in low-income groups. Prior authorization unduly burdens routine diagnostics, routine interventional and surgical procedures, and routine cardiac specific medication use in the ACPC population. This manuscript highlights the burdens of prior authorization and advocates for the elimination of prior authorization for ACPC patients.

## Introduction

Prior authorization is a process that health insurance companies use to determine if a patient’s health insurance policy will cover certain medical treatments, procedures, or medications. The process typically involves the healthcare provider submitting a request to the insurance company, which then evaluates the request based on specific criteria, such as medical necessity and coverage policies. Prior authorization is a payer-initiated process to assess whether a proposed service or treatment is medically indicated [1]. In the 1960s, as an early model of prior authorization, Medicare and Medicaid created a health insurance costs and utilization review system with the goal of reducing overutilization and waste [2]. However, prior authorization’s use in the modern era has resulted in substantial administrative burden, unnecessary delays in patient care, and care-delivery inequities [3]. The medical community has repeatedly expressed that these hardships outweigh the financial gains for payors, and that the added administrative work drives increased overhead and physician burnout [4, 5].

In 2016, the American Medical Association surveyed physicians (40% primary care and 60% specialist) about their perspectives on prior authorizations. About 75% of physicians described the burden of prior authorization on physicians and staff as high or extremely high. Almost 25% of practices reported spending over 20 hours per week processing prior authorizations. 90% of providers reported a care delay associated with prior authorization even though 79% of claims were ultimately approved. Moreover, 34% of offices had staff members whose sole responsibility was to process prior authorizations [4]. In 2017, The American College of Cardiology published a survey of its membership that demonstrated not only administrative costs and delays in care, but also major safety concerns associated with the prior authorization process [5]. 77% of cardiologists felt there was less time spent on patient care by the practice, clinician, or staff because of the time required for medical documentation and the prior authorization process. 62% felt that this resulted in significant patient confusion and treatment interruption. Additionally, 78% of denials conflicted with the American College of Cardiology’s (ACC) published Appropriate Use Criteria. The lack of agreement between insurers and providers erodes the patient’s and family’s trust in the healthcare system.

Previous studies have raised concerns about exacerbations of social inequities that are the result of the prior authorization process. A study of 161,181 patients found that prior authorization rejections were more common for women, racial minorities, those with low education, and in low-income groups [3]. Another study of 16,853 qualified health plans in the 2019 Affordable Care Act Health Insurance Marketplace, found that prior authorization rate for HIV pre-exposure prophylaxis was 37% in the South compared with 13% in the Midwest, 6% in the West, and 2% in the Northeast [6]. The impact of prior authorization on under resourced and/or low-income individuals is especially concerning as prior authorizations have been found to paradoxically increase medical costs by creating non-adherence, worsening disease states, and increasing hospitalization rates [7].

In addition, other studies show that although prior authorization denials within pediatric subspecialties remain low, they still lead to significant administrative burden, and can lead to potential harm. Denial rates in pediatric hematology and oncology are low at 1.5%, but they require an average of 46 minutes of staff time per denial to process [8, 9]. Prior authorization denial rates in pediatric emergency medicine are only 3%, but the administrative burden remains substantial [10]. Delays in treatment initiation have been found in pediatric neurology for anti-epileptics [11] and in pediatric gastroenterology for inflammatory bowel disease [12]. Requiring prior authorization for tonsillectomy showed a denial rate of 1.5%, no change in the overall incidence of tonsillectomy surgery, and an associated delay of 2.38 days from consultation to surgery [13]. These prior authorization processes provide additional stress to an already challenging economic environment for Children’s Hospitals. Lack of prior authorization reform within pediatrics may contribute to the trend of closures in US hospitals [14, 15].

Caring for patients with complex congenital heart disease requires collaborative subspecialty knowledge, specialized and time-intensive diagnostic imaging, unique interventional and surgical procedures, and congenital cardiac specific medications. The heterogenous nature of congenital heart disease makes management less algorithmic and more patient-disease specific. Perhaps unsurprisingly, payor’s prior authorization algorithms have been designed to address the adult cardiology population who have the highest volume of mostly homogenous claims. A 2019 paper published in the Journal of the American Medical Association revealed that there are over 10 times the number of adult cardiologists (30,000) than pediatric cardiologists (2900) [16]. As per the CDC, there are about 20.1 million adults with coronary artery disease which is the most common diagnosis for the $229 billion spent in adult cardiology [17]. Whereas the adult congenital and pediatric cardiology (ACPC) population only represents about 2.4 million patients and $6 billion [18]. The ACPC community is a small group within cardiology, and we suggest that this group deserves separate consideration. Prior authorization represents such a significant care burden that patient and family congenital heart advocacy organizations have created patient and provider guides to assist with the prior authorization process [19]. ACPC care should not be subject to the same prior authorization process and algorithms that were developed primarily for acquired disease in adult cardiology.

## Prior Authorization Burden with Routine Diagnostics

Patients with congenital heart disease all require routine diagnostics, but the required frequency for specialized diagnostics varies widely based on diagnosis. For many patients, an echocardiogram and electrocardiogram are indicated every time they attend clinic. Additionally, while the American Heart Association and ACC published guidelines for follow-up of adults with congenital heart disease in 2018, insurance company algorithms for prior authorization may not match the most recent data [20]. Congenital echocardiograms are acquired using fundamentally different protocols from adult echocardiograms, often requiring a physician to assist the sonographer and acquire images themselves [21], and this additional difficulty has been recognized by the creation of separate CPT codes. Cross-sectional imaging with CT, MRI, or 3D reconstruction are often necessary for routine surveillance and previous literature has commented on the difference in time and resource requirements to obtain these images (e.g. requiring anesthesia for sedated MRI, requiring that the cardiologist be present during the imaging in order to protocol in real-time for image acquisition), which is different than acquired disease in adult cardiology [22].

We retrospectively reviewed prior authorization practices at a single center ambulatory pediatric cardiology practice in Texas, using an administrative database. Review of over a thousand sequential clinical encounters by one of the authors (KS) found that 15% of echocardiograms required prior authorization, but the ultimate denial rate was less than 0.5% (Table 1). Prior authorization was required for 86% of MRI with about 10% requiring peer-to-peer interactions though none were denied. Experience with requests for CT imaging was similar, with 78% prior authorization and a low denial rate. These high rates of required prior authorizations coupled with the low rate of denial raise concerns about the value of prior authorization within ACPC diagnostics.

**Table 1:** Review of prior authorizations claims at an ACPC ambulatory practice

Service	Claims (number)	Prior authorizations (number, percent)		Denials (number, percent)		Peer-to-peer (number, percent)	
Echocardiogram	1276	196	15%	6	0.5%	4	2%
MRI/MRA	14	12	86%	1	7%	2	17%
CTA	9	7	78%	0	0%	0	0%
EP procedures	12	3	25%	0	0%	1	33%
Cath procedures	15	5	33%	1	7%	1	20%

## Prior Authorization Burden with Routine Interventional and Surgical Procedures

One in one hundred babies is born with congenital heart disease and one in four of those babies born requires surgery or catheter-based intervention within the first year of life [23, 24]. Diagnostic and interventional catheterization, invasive and non-invasive electrophysiology, and cardiac surgical procedures all require interdisciplinary teamwork between cardiology, interventional cardiology, electrophysiology, cardiac surgery, and cardiac anesthesia. Each of these services may require its own prior authorization for their role in the care plan of a congenital heart patient. Additionally, unlike adult cardiology, where a procedure (such as coronary angiography) is associated with a homogenous patient group suffering from atherosclerotic disease, in ACPC coronary angiography is utilized in the management of a wide variety of diseases. Further complicating matters is the potential discovery of a new lesion during pre-surgical evaluation, which would require a second round of prior authorizations. The disjointed nature of the prior authorization process and repeated needs for prior authorization to manage a single patient creates delays in care for procedures that likely affect patients’ morbidity and mortality and may be used by payors as a reason to deny payment for procedures that were not previously approved.

## Prior Authorization Burden with Routine Cardiac Specific Medications

ACPC providers cope with several challenges in medication prescriptions given the nature of pediatric pharmaceutical development and distribution. Each of the following issues is a common cause for prior authorization. Given the heterogeneity in the ACPC population, there is substantial difficulty in generating high-quality guidelines similar to our adult cardiology colleagues to guide payors. These challenges partly stem from lack of financial incentive for the pharmaceutical industry to conduct adequately powered clinical trials, changes in age related physiology in pediatric patients, and ethical considerations with pediatric research [25].

## Compounded Medications

Medications are commonly unavailable in an appropriate dosage form in pediatrics [26]. Children rely heavily on compounding pharmacists to supply their medications. Commonly compounded medications in pediatric cardiology include spironolactone, sildenafil, propranolol, nifedipine, hydralazine, furosemide, losartan, digoxin, and captopril [27] as well as most anti-arrhythmic medications for newborns including ivabridine, amiodarone, carvedilol, and flecainide.

Several medications (e.g. enalapril) have more recently become available in pediatric solutions; however, the new formulation is sold at higher costs. This new formulation has led to a change in coverage by several payors, with such new formulations now considered Tier III drugs. The transition from compounded medications to the new expensive formulations has somewhat counterintuitively made these therapies less available to patients and unsurprisingly has been accompanied with an arduous prior authorization process. Tier III drugs are rarely approved by state Medicaid, which contributes to horizontal inequity in congenital heart disease. Barriers with compounded medication often lead to discharge delays and increase the likelihood of missed doses as outpatient.

## Off-Label Medications

Off-label medications are commonly used in pediatrics [25, 28]. Previously published data have indicated that roughly 70% of all cardiovascular medications administered in congenital cardiology are off-label. Of 31,432 patients included in the study, 75% received at least 1 cardiovascular medication off-label, and 31% received at least 3 cardiovascular medications off-label [29].

Sildenafil was only recently approved in children after extensive post-market clinical trials [30]. Bosentan, and ambrisentan (commonly used pulmonary vasodilators) are still not approved for pediatric patients, though are routine and evidence-based standard for ACPC patients with pulmonary hypertension. Direct oral anticoagulants (including rivoraxaban and apixaban) continue to be used off-label in pediatrics given hardships with warfarin in the pediatric population. Perhaps most striking is the fact that carvedilol, a beta blocker used ubiquitously in pediatric heart failure, is used off-label in pediatric patients. In recent years, adult cardiology has made substantial advances in medical therapies with many new heart failure and cardiomyopathy medications. To apply these therapies to the pediatric population requires off-label prescribing. Because insurance companies commonly require prior authorization for off-label medications the prior authorization process unduly burdens pediatric patients [31].

## Payor Formularies Do Not Match Evidence-Based Practice

Another common concern in ACPC occurs when an evidence-based recommendation does not match a payor’s preferred formulary. This occurs specifically in Long QT syndrome, where multiple manuscripts have demonstrated superiority of nadolol [32, 33] compared with other beta blockers. Unfortunately, nadolol is minimally used in adult medicine and accordingly, while the medication itself is not expensive, nadolol is often not on preferred formularies. This disconnect between the formularies and the medical evidence is also seen with rosuvastatin for hyperlipidemia. The PULSAR study clearly demonstrated benefit of rosuvastatin over atorvastatin, yet claims are still denied for rosuvastatin [34]. Evidence-based practice should not require prior authorization.

## Vulnerable Patient Groups

Patients with single ventricle physiology, pulmonary hypertension, heart failure, and heart transplant are specific groups within ACPC that require additional attention because of their lifelong high burden of care. These complex congenital heart patients are typically on several medications lifelong that require annual prior authorization, which, ensures that prior authorization is a lifelong hardship that follows these complex patients. These patients are also often those who suffer the greatest clinical fragility, debility, and challenges with maintaining insurance coverage in adulthood. Moreover, these vulnerable populations are significantly impacted by longstanding systemic inequities and disparities in the social determinants of health [35]. Creating medical systems that facilitate equitable clinical care is paramount to their quality of life for patients with complex congenital heart disease.

## Proposed Solutions

**Fig. 1:**
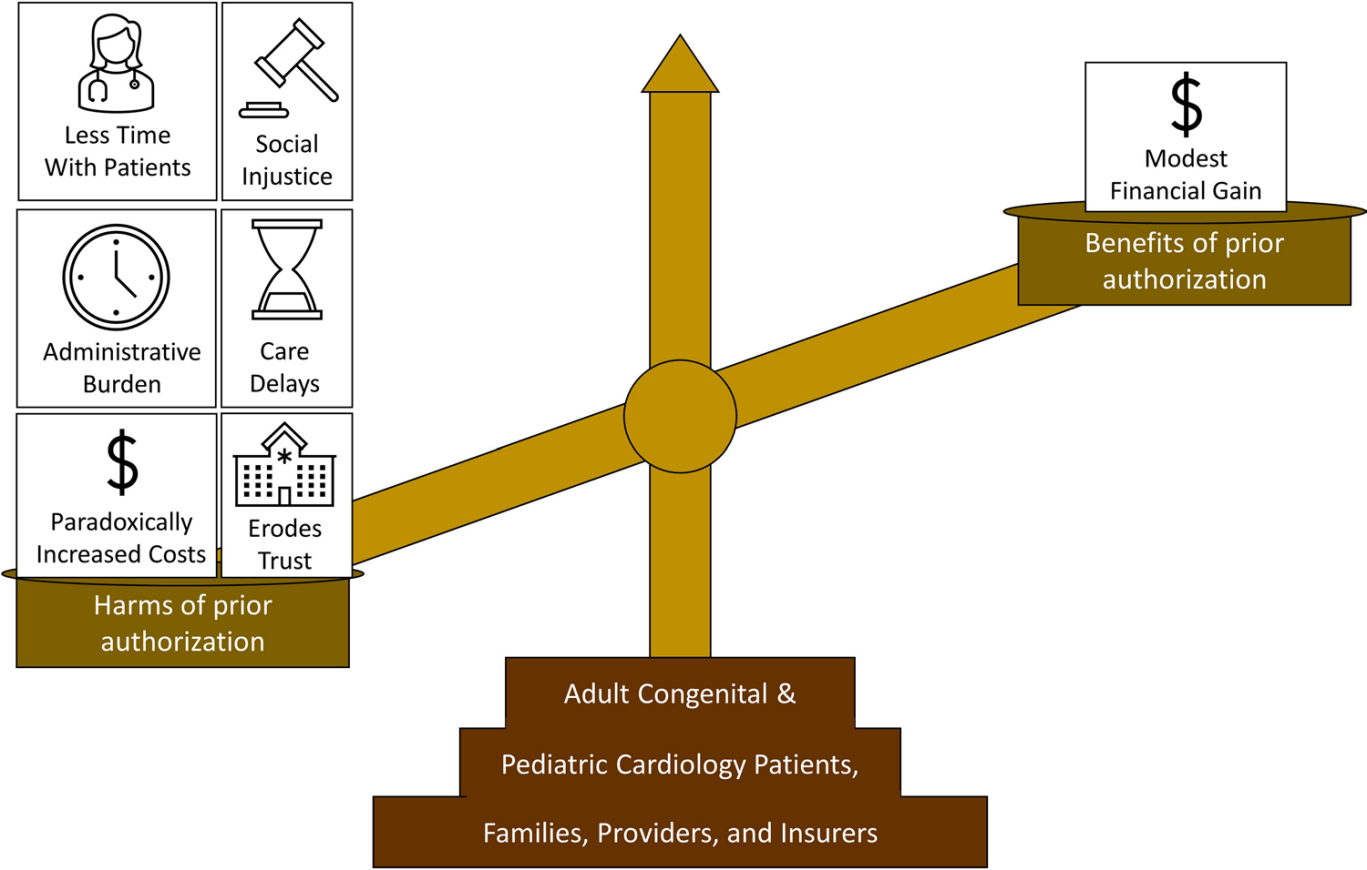
All parties benefit from eliminating prior authorization in pediatric and adult congenital cardiology

We advocate for the discontinuation of prior authorizations for routine diagnostics, all congenital heart disease related interventional and surgical procedures, and routine cardiac disease specific medications for the ACPC patient population. Requiring prior authorizations for a small, complex subspecialty such as ACPC confers harm in the form of administrative burden, care delays, and worsening social inequity without evidence of significant financial benefits (Fig. 1). Anecdotal reports, studies from other pediatric subspecialities, and our single center analysis shows the denial rate is close to zero. Steps have already been taken at the legislative level to eliminate the prior authorization process in certain settings. In Texas, House Bill 3459 passed in 2022 so that providers with a 90% or higher prior authorization approval rating will be exempt from future prior authorization burden for that service in the future [36]. In Maryland, Senate Bill 688 was introduced to require insurers adhere to the 48 hour turnaround time for prior authorization claims and eliminated prior authorizations for generic medications [37]. These bills are important steps toward appropriate change in payor-reform, but larger and broader changes must be implemented such as requiring public reporting of denial rates and care delays related to prior authorizations or, better yet, eliminating the prior authorization process altogether. Once prior authorization is eliminated, providers can use guidelines published in peer-reviewed journals to guide appropriate use as we continue to research the optimal diagnostic and treatment strategies for this complex patient population.

## Conclusion

Prior authorizations for patient care of the ACPC patient population are commonly required, but prior authorization is rarely denied. As a consequence of providing care to complex, resource-intensive patients, the physicians providing care to this population are subjected to an administrative burden without benefit to the payors, patients, families, or medical professionals. The protocols created for these prior authorizations are largely based on adult cardiology practice, which has created a system that unduly burdens ACPC patients and providers and, ultimately, jeopardizes patient care. We assert that the administrative burden, care delays, and concern for worsening social inequities caused by prior authorizations far outweighs whatever the small financial benefit to the payor from the rarely denied claims may be. An effective and cost saving approach may be simple: discontinuation of prior authorizations for diagnostics, interventional and surgical procedures, and cardiac disease specific medications for the ACPC population.
